# Ethnobotany of the Balti community, Tormik valley, Karakorum range, Baltistan, Pakistan

**DOI:** 10.1186/s13002-016-0114-y

**Published:** 2016-09-09

**Authors:** Zaheer Abbas, Shujaul Mulk Khan, Arshad Mehmood Abbasi, Andrea Pieroni, Zahid Ullah, Muhammad Iqbal, Zeeshan Ahmad

**Affiliations:** 1 Department of Botany, Hazara University, Mansehra, Pakistan; 2 Department of Plant Sciences, Quaid-i-Azam University, Islamabad, Pakistan; 3 Department of Environmental Sciences, COMSATS Institute of Information Technology, Abbottabad, Pakistan; 4 School of Light Industry and Food Sciences, South China University of Technology, Guanzhou, China; 5 University of Gastronomic Sciences, Bra/Pollenzo, Italy; 6 Center for Plant Sciences and Biodiversity, University of Swat, Swat, Pakistan

**Keywords:** Ethnobotany, Medicinal plants, Indigenous knowledge, Karakorum, Pakistan

## Abstract

**Background:**

Limited health facilities and malnutrition are major problems in the Karakorum Range of Northern Pakistan, often resulting in various human disorders. Since centuries, however, local communities in these areas have developed traditional methods for treating various ailments and local foods capes that can be significant for devising public health and nutritional policies. This study was intended to document the ethnobotanical knowledge of the local peoples in the Tormik Valley, especially in the medical and food domains.

**Methods:**

Field trips were undertaken in 14 different villages of the study area from 2010 to 2012. Ethnobotanical data were gathered using semi-structured interviews and group conversation with 69 informants. Details about local uses of plant species were recorded along with demographic characteristics of the visited communities. Relative frequency citation index (RFCi) and preference ranking index (PRi) tools were applied to determine the cultural significance of the reported species.

**Results:**

Sixty-three plant species, with a predominance of Asteraceae and Fabaceae family members, as well as their detailed folk uses were documented. Forty-three percent of the species were used to treat various diseases, 21 % were consumed as wild fruits and vegetables and 53 % of the species had multipurpose applications. *Thymus linearis* Benth, *Hippophae rhamnoides* ssp. *turkestanica* L. and *Convolvulus arvensis* L. were found to be the most utilized medicinal plant species, i.e. those with significant RFCi values (0.54, 0.51 and 0.48, respectively). *Betula utilis* D. Don was the most versatile taxon (seven different ways of utilization); being this species a common and easily accessible subalpine tree and then under anthropogenic pressure, the implementation of concrete strategies aimed at its in-situ and ex-situ conservation is strongly recommended.

**Conclusion:**

The valleys in the Karakorum Mountains in the Northern Pakistan host significant Traditional Knowledge on local food and medicinal plant species, which need to be reconsidered and cautiously re-evaluated by ethnopharmacologists, and public health/nutrition actors. Furthermore, germane trans-disciplinary investigations are suggested to ensure the dynamic conservation of precious local knowledge systems, as well as plant diversity in Pakistani mountain regions.

## Background

Human beings have been using plants since ancient times for many purposes and early on they especially developed several ways of using plant resources in order to counteract diseases [1, 2]. Many field studies in the last decades have shown that traditional peoples, local communities, and indigenous societies around the world retain a tremendous local plant knowledge, remarkably embedded into daily practices and mainly orally transmitted [3, 4].

Natural resources and associated biological diversity provide the basis of livelihood for human populations. Consequently, humans have a great impact on local vegetation and vice versa [5]. Ethnobotany is the burgeoning interdisciplinary scientific field which covers all sorts of interactions and relationships between plants and people. The history of medicinal plant use by humans to treat diverse ailments dates back to ancient civilizations [6, 7]. Even though the advent of allopathic medicine has somehow minimized the role of medicinal plants in favor of synthetic drugs, a number of modern drug discoveries have been based on medicinal plants used by indigenous peoples [8]. In Pakistan approximately 6000 species of higher plants are found [9, 10]. At least 12 % of the flora species are used medicinally, several of which are exported [11]. Of these species, the active constituents of approximately 500 species are known. Diversity in plants and variations in plant-people interactions are further influenced by the selection of wild plants for food and other native cultural uses. One of the primary objectives of ethnobotanical investigations is the documentation of indigenous knowledge associated with these plant species which is diminishing day by day in general, and among people living in close proximity to the forest in particular [12].

The people of remote areas in any region rely on local resources in order to treat various health disorders [13–15]. Ethnobotanical information can provide an important feedback for public health and environmental policies through the understanding of socio-cultural backgrounds and the analysis of ethnic-based strategies to combat diseases [16]. In mountainous ecosystems such as the Karakorum range, often inadequate nutrition remains a major problem resulting in various diseases. The local inhabitants in these areas have developed traditional methods of curing such common health problems, which in turn can provide important data for devising public health policies [17]. The Karakorum mountain range, situated at the junction of western and central Asiatic regions of Tethyan flora, is one of the most diverse habitats in the world [18]. The Baltistan province of Pakistan is home to more than a dozen geographically isolated and botanically unexplored valleys in the Karakorum Range [19]. Although a number of previous ethnobotanical investigations have been conducted in surrounding areas [13, 14, 18–24], many of these studies did not use quantitative methods [9]. Moreover, Tormik Valley repeatedly went unnoticed, perhaps due to its high altitude, harsh and hostile climate, inaccessibility and prevailing poverty. A large proportion of its inhabitants depend on herbal remedies. They are known as the trustees of cultural knowledge whether related to plants, animals, fungi, lichens, or stones. However, no in-depth ethnomedicinal survey in this valley has been conducted thus far. Therefore, the current study records and documents the medicinal uses of plant species by the inhabitants of the region aiming to: (i) document traditional knowledge of plant species used by the Balti communities; (ii) quantify the ethnomedicinal plant uses employing relative frequency of citation (RFC) and preference ranking (PR) indices; (iii) highlight the most interesting and novel medicinal plants that have never previously been reported in nearby areas and whose phytochemistry and pharmacology should be further investigated.

## Methods

### Study area

#### Geography

Tormik Valley is situated on the right bank of the Indus River in Baltistan District, in the Karakorum mountain range, of Northern Pakistan. The valley covers a land area of 2750 km^2^, of which about 1010 km^2^ (36 %) encompasses natural pastures, at an elevation range of 2000–6000 m asl. Despite being a narrow valley, it is home to 27 permanent villages (main villages) and sixteen temporary summer settlements (sub-Alpine settlements) situated on the banks of the Tormik River [19]. Floristically, the valley belongs to the eastern Irano-Turanian sub region [9].

#### Climate and topography

The climate is characterized by a prolonged and hostile winter with repeated snowfall which severely restrains daily activities. The lower terrain is rugged, stony and exhibits a dry desert environment, but at higher elevations frequent precipitation gives rise to relatively richer vegetation. No weather station exists in the region; however, data from Skardu, which is 55 km away, shows a mean monthly temperature of 11.5 °C, with a winter minima of −23.2 °C and a summer maxima of 41 °C [20].

#### Ethnographic background

Overall in the Baltistan region (province), Mongol, Mon, Hor, Brokpa and Kashmiris are the prominent ethnic groups [21] with the local languages being Balti and Shina (Broq-skat); however, the studied valley hosts a single ethnic group: the Balti. This ethnic group is comprised of thirty-one lineage groups known as *qoum* and speaks Balti as their local language. The population of the valley is approximately 5,000 inhabitants [22] comprising 706 households. The people of this region migrated to the study area from other parts of Baltistan, as well as other regions, before the birth of the founder of Buddhism, Guatama Budha (563 BC) [23, 24].

#### Socio-economic profile

A large proportion of the valley population is very poor and depends upon agriculture, livestock rearing, and the production of fuelwood, wool blankets (Qaar), gemstones, and thatched goods (baskets and grass holders), as well as other forest resources. There is no formal marketing of medicinal plants which indirectly benefits herbal businessmen (middle men) rather than customers who are the real custodians.

### Ethnobotanical data collection

**Fig. 1:**
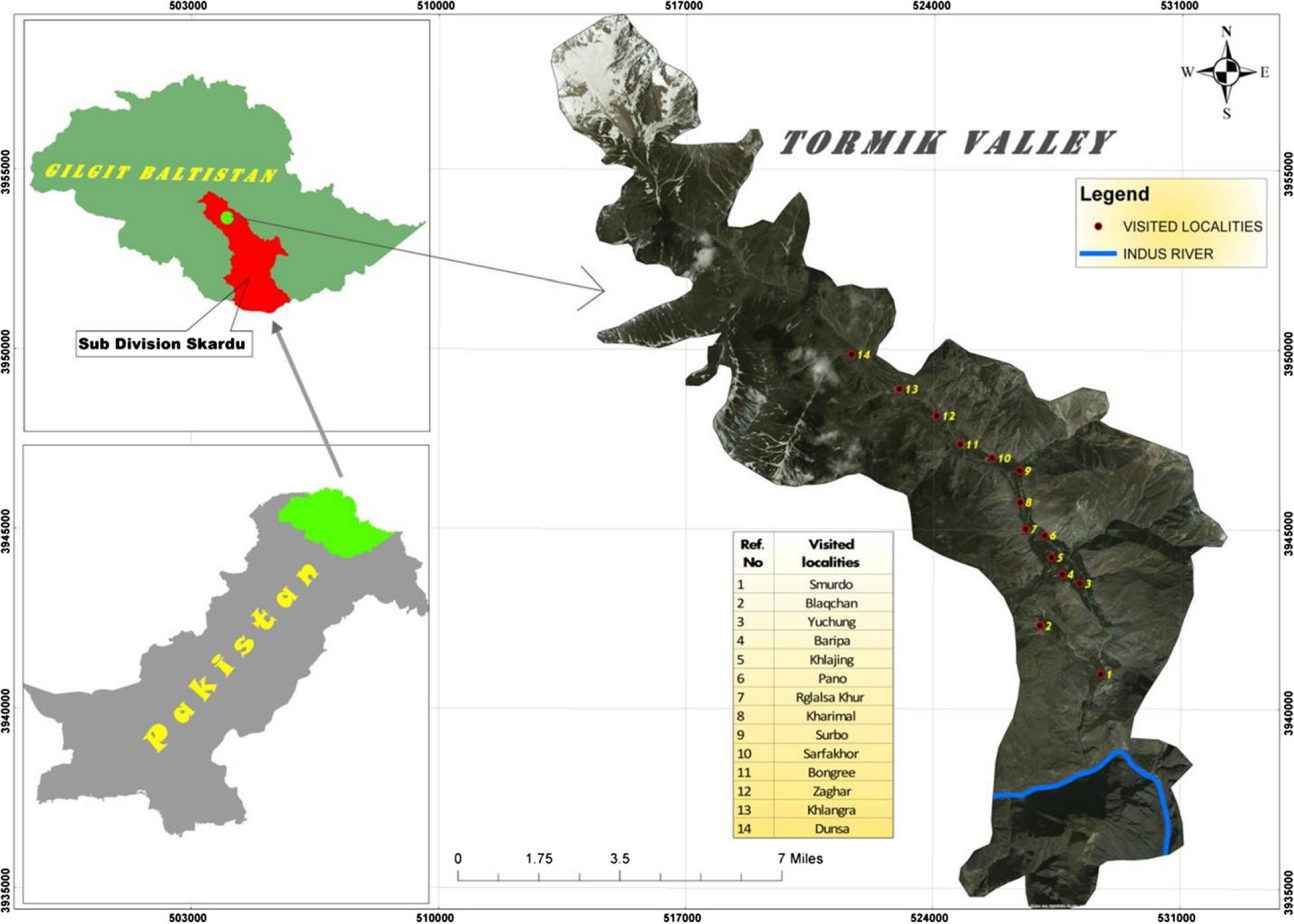
Map showing the study area and the visited localities

Ethnobotanical data was collected from 69 different informants (including 37 males, 27 females and five local herbalists) in the valley villages of Smurdo, Blaqchan, Yuchung, Baripa, Khlajing, Pano, Rgialsakhor, Harimal, Surbo, Sarfakhor, Bongree, Zaghar, Khlangma and Dunsa from 2010 to 2012 (Fig. 1). Formal consent was received from informants regarding data collection and publication; then the Participatory rural appraisal (PRA) approach as mentioned in the Kyoto Protocol was applied with the consent of the informant. Ethical guidelines of the International Society of Ethnobiology (http://www.ethnobiology.net) were strictly followed. The methodology was designed with the sole purpose of obtaining the invaluable wealth of local knowledge, with special emphasis on medicinal plant use [21, 25]. The informants were classified into eight age groups, i.e.: 11–20, 21–30, 31–40, 41–50, 51–60, 61–70 and above 70 years of age (Tables 1 and 2). Questions regarding the vernacular name, availability, part(s) used, modes of preparation and administration, diseases treated, and cultural uses were asked in the Balti native language, in which the first author is fluent. Within the chosen sample, women often disliked speaking with strangers due to their isolated social organization and religious teachings. Therefore, female informants always had to be introduced through their male relatives (e.g. husband, father or brother) [25].

**Table 1:** Characteristics of the study participants

Categories	Number	Percentage
Gender		
Men	27	39.13
Women	42	60.87
Total	69	
Age group		
Below 50 years	20	28.99
Above 50 years	49	71.01
Education level		
Illiterate	46	66.67
Primary	5	7.25
Middle	5	7.25
High school	9	13.04
Graduate	2	2.90
Masters	2	2.90
Socioeconomics		
Farmers	19	27.53
Shepherds	5	7.25
Hunters	5	7.25
Wood cutters	8	11.59
Teachers	5	7.25
House wives	27	39.13

**Table 2:** Total number and percentage of interviewees per age group

Age group	No of interviewees	Male	Female	Percentage
11–20	3	2	1	4.35
21–30	6	5	1	8.70
31–40	4	2	3	5.80
41–50	7	4	2	10.14
51–60	20	9	11	28.98
61–70	23	3	20	33.34
Above 70	6	2	2	8.70

Collected plant species were identified using the *Flora of Pakistan* and other literature sources [26]. The botanical names and respective families were confirmed via the *Angiosperm Phylogeny Group* [27]. Collected specimens were given voucher numbers and stored in the Herbarium of Quaid-i-Azam University, Islamabad, Pakistan.

### Data analysis

Data were analyzed based on use reports of plant part(s) of each species to treat a given health disorder. Traditional remedies of each taxon along with diseases cured were evaluated using the number of citations by the informants. The significance of each plant species was estimated by the relative frequency citation index (RFCi), which indicates the local importance of each species. RFCi values were calculated via the frequency of citation (FC, the number of informants mentioning the use of the species) divided by the total number of informants participating in the survey (N), without considering the use-category values as reported previously [28–30]. The preference ranking index was calculated, as explained by Asase et al. [31], according to the level of effectiveness of the reported plant species. Each rank was given a numeral (1, 2, 3, 4 or 5), with the most effective plants assigned a value of 5.

## Results and discussion

This work helps to understand traditional ecological knowledge, which now also includes an analysis of how this knowledge is adapted, linked, and transmitted through generations [32].

### Informant demographics

Elderly informants, who were mostly farmers, shepherds, wood cutters, teachers, tourist guides and housewives, have more ethnobotanical knowledge compared to younger ones. This might be due to changing lifestyles, the urbanization of towns, a greater dependence on allopathic medicines and the lack of interest by younger generations. Two-thirds of the informants were illiterate due to the lack of education facilities, while the remaining one-third were educated (mostly secondary school level or below) (Table 1).

### Taxonomic diversity

According to Dickoré [33], the Karakorum Range exhibits a wide array of landscape patterns and a diverse ecology supporting a unique composition of flora. The flora of the Karakorum Mountains is poor in species number, but dominated by taxonomically complex groups. In total, 63 plant species belonging to 32 families and 55 genera were documented as used by the Balti community (Table 3). The two most important families were Asteraceae and Fabaceae with 7 species each, followed by Rosaceae with 6 species in terms of ethnobotanical usage. Six families were represented by two species and 20 families by only a single species each. Among genera, *Artemisia, Astragalus, Juniperus* and *Trifolium* each featured two ethnomedicinally important species. With respect to growth habit, herbs were the dominate form (88 %), followed by trees (8 %) and shrubs (4 %).

**Table 3:** Folk medicinal plant uses recorded in Tormik Valley, Karakorum Mountain Range

Latin name/family/voucher number	Local name	Parts used	Drug description	Diseases treated	RFCi (*n* = 69)	PRi
*Allium carolinianum* Redoute. Alliaceae QAU 127126	Chong	Bulb	A fresh bulb decoction is taken three times a day while a bulb paste is applied topically on painful joints and bones	Gastrointestinal disorders, bone or joint pain	0.17	1
*Artemisia scoparia* Waldst. & Kit. Asteraceae QAU 127156	Khobustae	Flower & leaves	The flowers and leaves are boiled and the decoction is taken twice a day	Abdominal worms, urethritis	0.20	2
*Berberis pseudumbellata* R. Parker Berberidaceae QAU 127186	Skiorbu	Flower, fruit, seed	Seeds and fresh fruits are eaten while a flower decoction is recommended three times a day	Jaundice	0.39	3
*Bergenia ciliata* (Haw.) Sternb. Saxifragaceae QAU 127281	Schapur	Rhizome	A decoction of rhizome is taken twice a day while a paste is applied topically on eyelids	Stomach ulcer, eye ache	0.30	3
*Cicer microphyllum* Royle. Fabaceae QAU 127253	Stranjungstwa	Whole plant	Fresh plants are collected and cooked in water as a vegetable. It is suggested the plant is eaten raw once a day	Kidney stones, urinary problems	0.32	3
*Convolvulus arvensis* L. Convolvulaceae QAU 127220	Thringthringmo	Whole plant	Fresh plants are boiled in water as a vegetable and eaten with wheat bread twice a day	Constipation	0.48	4
*Cousinia thomsonii* C.B.Clarke. Asteraceae QAU 127162	Charchu	Flower	The flower is boiled in water and applied topically on infected areas as needed	Dermatitis	0.14	1
*Delphinium brunonianum* Royle Ranunculaceae QAU 127278	Makhoting	Whole plant	The whole plant is dried and ground with water and the paste is then applied on the head as hair tonic	Hair tonic	0.36	3
*Descurainia sophia* (L.) Webb ex Prantl Brassicaceae QAU 127197	Khashir	Whole plant	A decoction of the whole plant is made and recommended thrice daily	Asthma, constipation	0.16	1
*Equisetum arvense* L. Equisetaceae QAU 127121	Thangshing stwa	Whole plant	A decoction of the whole plants is taken twice daily	Urinary tract disorders	0.28	1
*Fagopyrum esculentum* Moench. Polygonaceae QAU 127266	Bro	Seed	The seeds are ground and the powder is taken with water three times a day as needed	Stomach ulcer, tumour, jaundice	0.45	4
*Hippophae rhamnoides* ssp. *turkestanica* L. Eleagnaceae QAU 127225	Karsoq	Fruit & leaves	A fresh fruit paste is taken twice daily while a decoction of leaves is taken twice a day/a leaf paste is rubbed on infected parts	Gastrointestinal disorders,dermatitis	0.51	5
*Hyoscyamus niger* L. Solanaceae QAU 127297	Landungstwa	Seed	The seeds are ground with water and the paste is applied on aching teeth and gums twice a day	Toothache	0.12	1
*Juniperus excelsa* M. Bieb. Cupressaceae QAU 127123	Shukpa	Fruit	The fresh fruits are boiled and the decoction is taken twice a day as needed	Stomach ulcer, fever	0.13	1
*Mentha royleana* Wall. Lamiaceae QAU 127139	Foling	Leaves	A decoction of leaves is made and taken three times a day	Abdominal pain & gastric problems	0.42	4
*Onosma hispida* Wall. & G.Don. Boraginaceae QAU 127193	Kangmar	Whole plant	The whole plant is cooked in water as a vegetable and taken twice a day as needed	Jaundice, constipation	0.25	2
*Pimpinella diversifolia* DC. Apiaceae QAU 127140	Kohniod	Whole plant	The whole plant is boiled in water and the soup is taken three times a day	Fever, stomach ulcer, as a blood purifier	0.26	2
*Pleurospermum candollei* (DC.) Benth. ex C.B. Clarke Apiaceae QAU 127141	Braqshundun	Whole plant	A decoction of the whole plant is made and taken twice a day	Jaundice	0.32	3
*Pulsatilla wallichiana* Ulbr. Ranunculaceae QAU 127280	Zgiongmonana Loqparimandoq	Flower	Dried flowers are ground and the powder is applied topically on infected skin twice a day	Dermatitis	0.13	1
*Rheum australe* D.Don. Polygonaceae QAU 127272	Shoot	Root	The fresh and clean root is boiled in water and the decoction is given three times a day	Asthma, fever, pneumonia	0.38	3
*Rumex nepalensis* Spreng. Polygonaceae QAU 127271	Rashona	Leaves	The leaves are first boiled and chopped; then the paste is applied on infected skin two times in a day	Dermatitis	0.19	1
*Solanum nigrum* L. Solanaceae QAU 127295	Drumbashokhlo	Fruit	The fruits are toasted and applied to aching teeth three times in a day	Toothache	0.23	2
*Tanacetum falconeri* Hook.f. Asteraceae QAU 127180	Tyalo	Whole plant	A decoction of the whole plant is recommended once a day	Body ache, fever	0.36	3
*Thymus linearis* Benth. Lamiaceae QAU 127243	Tumbruk	Flower	Flowers are boiled in water and the decoction is taken twice daily	Abdominal pain, vomiting	0.54	5
*Tribulus terrestris* L. Zygophyllaceae QAU 127310	Kokoring	Whole plant	A decoction of the whole plant is taken orally twice a day, whereas for body itching the decoction is used as a bath	Urinary disorders,body itching	0.33	3
*Urtica dioica* L. Urticaceae QAU 127308	Khaeshing	Whole plant	The whole plant is boiled in water and the decoction is taken orally thrice daily, whereas boiled and chopped leaves are applied on pimples and pustules	Joint pain, blood tonic, pimples	0.28	2

*RFCi* relative frequency citation index, *PRi* preference ranking index

**Fig. 2:**
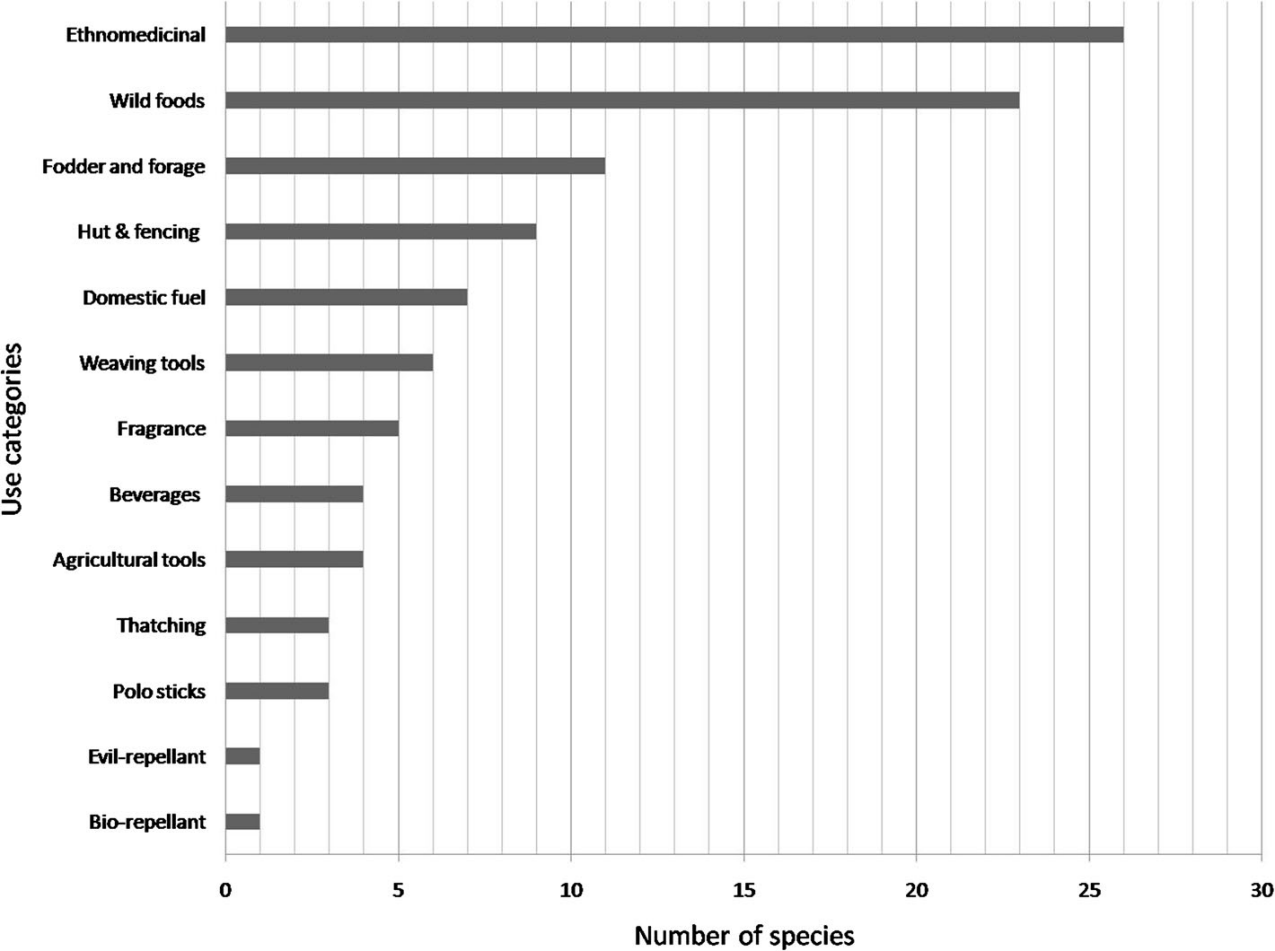
Ethnobotanical use categories of plant species

Reported species were classified into three main classes on the basis of their nature of usage, i.e., ethnomedicinal (26 spp., 43 %), wild edible including fruits and vegetables (23 spp., 21 %), and cultural plants (34 spp., 53 %). Furthermore, cultural plants were further categorized into 11 use categories, i.e., beverages (4 spp.), hut and fencing (9 spp.), thatching (3 spp.), agricultural tools (4 spp.), domestic fuel (7 spp.), fodder and forage (11 spp.), wool weaving tools (6 spp.), bio-repellant (1 sp.), evil repellant (1 sp.), fragrance and affection (5 spp.), and polo stick making (3 spp.) (Fig. 2).

### Vernacular nomenclature

Vernacular nomenclature represents the local names of plant species used for medicinal or cultural purposes. In some cases the local names of plant species provide clues about myths, social associations, habitat type, growth habit, stem structure, fruit and medicinal uses. For instance, the vernacular name of *Equisetum arvense* L. is *thangshing stwa* (from *thangshing: Pinus* and *stwa:* grass) which means “*Pinus* like grass”. This name has been given due to the similarity in leaf shape between these two taxa. *Solanum nigrum* L. is referred to as *drumba shokhlo* (from *drumba*: homegarden and *shokhlo*: type of grape), as its fruit (berry) resembles grapes (*Vitis* spp.). *Pleurospermum candollei* (DC.) Benth. ex C. B. Clark. grows in rocky habitats of the study area, and as a result it is called *braq shunadun* (from *braq*: rocky, type of *shundun* which grows in rocky areas). *Pulsatilla wallichiana* Ulbr. has a rather long name locally, namely *Zgiongmo-nana-loqpari-mandoq*. Interestingly, it refers to the relationship of a daughter-in-law and her mother-in-law. In the Baltistan region, particularly in rural areas, people live in joint families. Due to the often hostile relationship between daughters-in-law and mothers-in-law, it has been a burning topic of the society, especially among women. Regarding the naming of this taxon, it is said that the flowers of the species are always seen opposite to each other in direction when they bloom, exemplifying the antagonistic relationship of these two ladies. Similarly, *Onosma hispida* Wall. & G.Don. is known as *kangma*r (from *kangma*: foot and *marfo*: red) which means “plant with red foot”. *Biowa-charchu* (from *biowa*: rat) is the local name of *Astragalus psilocentros* (L.) A.Gray, as this species is used to stop rats from nesting in homes, stores, cattle barns, etc. *Chenopodium foliosum* Asch. is called *spang-osae* (from *spang*: grassy habitat and *osae*: mulberry) indicating the type of mulberry which grows among grasses. This name has been assigned to the species given its resemblance to the mulberry tree (*Morus* spp.) in the shape of its fruit. According to the local people, *Codonopsis clematidea* C.B. Clark is eaten extensively by sheep. Therefore, it has been given the name *loo-sunma* (from *loo*: sheep and *sunma*: vegetable) which means “the vegetable of sheep”. However, the etymology of many other local names of plant species was not known; for instance, via for wild rose or *rhringthringmo* for *Convolvulus arvensis*.

### Ethnomedicinal uses

**Fig. 3:**
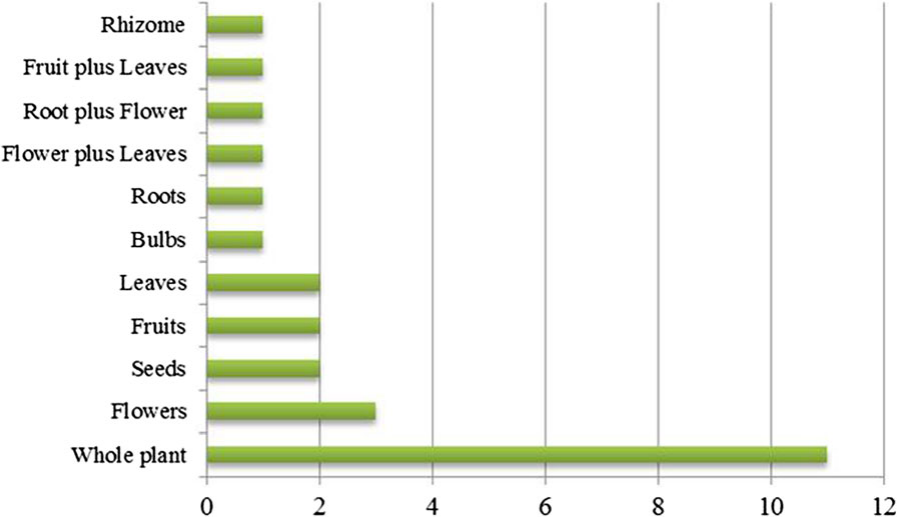
Number of ethnomedicinal uses of various plant parts

**Fig. 4:**
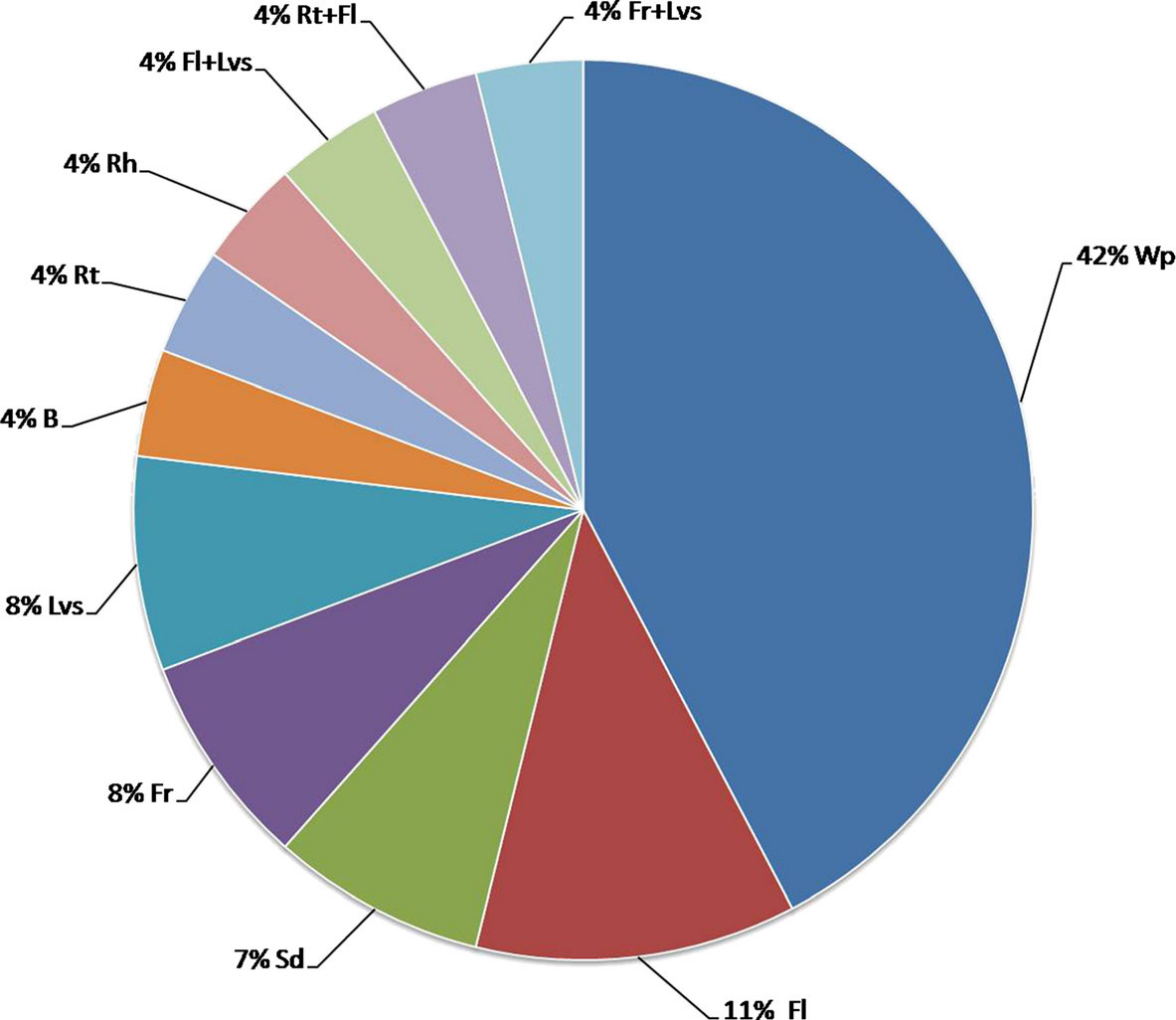
Classification of plant species based on part(s) used in traditional medicines. Wp: whole plant, Fl: flower, Sd: seed, Fr: fruit, Lvs: leaves, B: bark, Rt: root, Rh: rhizome, Fl + Lvs: flower and leaves, Rt + Fl: root and flower, Fr + Lvs: fruits and leaves

Traditional Medicines (TMs) are used worldwide and hence have global economic importance [25]. In developing countries, TMs are often the only accessible and affordable source of treatment [34]. Therefore, herbal remedies are the world’s therapeutic means to combat diseases for a large proportion of people in developing countries, in both rural areas and urban centers [27]. A total of 26 species representing 26 genera and 26 families were used to treat 11 different human ailments, namely gastro-intestinal diseases, dermatitis, jaundice, hepatitis, cancer, pneumonia, tonic, asthma, urinary disorders, joint pain and eye pain (Table 3). Recorded plant species were also examined for part(s) used, remedy preparation, route of administration and given dosage for a particular ailment. Eleven different parts of these 26 plants were being utilized in the region (Figs. 3 and 4). Regarding routes of administration, 62 % of species were taken orally and 15 % of species were reportedly applied externally, while 23.07 % were used either orally or externally.

#### Comparative analysis of our findings with previous works

The present study is the first ever comprehensive ethnobotanical investigation of Tormik Valley. However, the findings of the current study were compared with those of previous ethnobotanical investigations carried out in neighboring regions, as well as other countries. This study revealed that most medicinal plants were herbaceous, followed by trees and shrubs. The dominant herbaceous medicinal species in the flora of Karakorum are similar to those reported by previous studies [28].

Majority of the mentioned plant species are confined to Tormik Valley due to its unique micro climatic conditions. Some plants were also found in the adjacent regions as well as countries like China. Our findings are in contrast to certain previous ethnobotanical studies in neighboring regions as well as to other parts of the world, where different plant species were reported with respect to their preferred use [29–35].

As far as ethnobotanical importance is concerned, the uses of certain medicinal plants are the most significant (Table 3), as there is zero similarity in uses with [36–38] and few similarities with [39, 40]. This study reveals that more than half of the medicinal plant species were reported for the first time from Tormik Valley regarding their use (Table 3) [14, 41].

Interestingly, indigenous knowledge of plants and their uses change with respect to geographic area and ethnic group. For instance, the roots of *Astragalus psilocentros* Fisch.are used to treat the flu and tooth aches in other areas of central Karakorum [42], but in Tormik Valley the whole plant is used to cover holes in the walls of homes and cattle barns to prevent rats or mice from entering. The comparative medicinal knowledge of the ethnobotanical flora of the study area reveals diverse regional uses in the valleys of the Karakorum and Himalayan belts. Powder made from the berries of *Juniperus communis* L. is rubbed on rheumatic and painful swelling as well as burnt as incense in homes in Astor valley [43], but in Tormik Valley the same species is used as fuelwood only. The fruits of *Juniperus excelsa* M. Bieb. are used to treat stomach ulcers and fever in our study area, but according to [44] they are used for urinary tract problems. In the same way, the whole plant of *Delphinium brunonianum* Royle is used as a hair tonic in the current study area, which was also reported by along with other uses (i.e., baldness, diarrhea, stomach ache) in the valleys of Central Karakorum National Park (CKNP). In India and the lesser Himalayas of Pakistan *Solanum nigrum* has folk value as a liver tonic, to alleviate indigestion, to relieve eye pain, and to treat skin infections [45], but in the current study area the same species is used to alleviate toothache only. A leaf and flower decoction of *Artemisia scoparia* Waldst. & Kit. is used to treat abdominal worms in the study area as well as in Swat (Utror and Gabral) Pakistan [46], but it is used as a purgative in Gujrat [47]. The bulb of *Allium carolinianum* Redoute. is found to be effective for gastrointestinal disorders, bone pain and joint ailments but [48] reported its use to treat flu, fever and cough in Khunjerab Hunza. In the study area *Thymus linearis* is used to alleviate abdominal pain and vomiting while in Astore [43] reported its effectiveness in treating abdominal worms. *Convolvulus arvensis* is used as both a wild vegetable and a medicinal species to cure constipation, as also described by [47]. Likewise the fruits of *Hippophe rhamnoides* are used to treat gastrointestinal disorders and a paste made from its boiled leaves cures skin diseases. However, Khan and Khatoon [28] reported that the same species is effective for treating cardiac diseases, cancer and stomachache. In “Ladakh”, the district of India bordering Baltistan, this plant is used to treat gynecological disorders, i.e. irregular menstrual cycles, amenorrhea or dysmenorrhoea [49], and to improve digestion [50]. *Pimpinella diversifolia* DC is one of the most common medicinal herbs for abdominal disorders, fever and blood purification and it is also used in the Lesser Himalayan region of Pakistan to alleviate gas problems and indigestion [13, 51]*.* In Baltistan jams, pills and powders of *Hippophae rhamnoides* ssp. *turkestanica* are now commercially produced. However, currently the distribution status of this species is badly affected by over utilization and the lack of community awareness about its sustainability. Its local and regionally varied medicinal uses indicate that inhabitants can easily access and utilize this species, which is well-distributed along the valley floor of the Karakorum Mountains.

### Relative frequency of citation and preference ranking

Various ethnobotanical tools such as relative cultural importance indices are used to measure cultural preference of plant species in a particular area. We used the Relative Frequency of Citation index (RFCi) and the percentage of people with traditional knowledge (PPK) to assess the ethnobotanical knowledge of the local informants regarding medicinal uses of the reported plant species; these results are presented in Table 3. From a medicinal point of view *Thymus linearis*, *Hippophae rhamnoides* ssp. *turkestanica* and *Convolvulus arvensis* exhibited significant RFCi values (0.54, 0.51 and 0.48, respectively). These species also have the greatest frequency of encounter and PPK values (53.6, 50.7, and 47.8 %), with resultant PR values of 5, 5 and 4, respectively. High RFCi values indicate a greater number of citations by informants. A flower decoction of *Thymus linearis* is used to treat abdominal pain and vomiting. Bano et al. [21] reported that in Skardu Valley an infusion of *T. linearis* is used to treat cough, cold, pneumonia and other respiratory disorders. Although these uses were different than those observed in our study, the RFCi value is the same for both areas.

The fruits and leaves of *Hippophae rhamnoides* ssp. *turkestanica* are used for medicinal purpose, and local inhabitants use the whole plant in making huts and fences. In Tormik Valley *H. rhamnoides* was ranked second with a 0.51 RFCi value. The same species has been reported as characteristic and the top ranked species in Skardu Valley (RFC = 0.9) as its fruits are extensively used to treat arthritis pain and cough, to relieve skin inflammation in eczema and as a remedy for heart problems, ulcers, jaundice and urinary disorders [52]. Abbasi et al. [13] reported that *Solanum nigrum* showed the highest frequency of encounter (58 %) and a corresponding PR value of 5 in the Lesser Himalayas of Pakistan. However, in the present study its PPK value was only 22.3 %. In light of the diverse medicinal uses and variation in RFCi values of the same plant species in the region, it can be hypothesized that these plant species have diverse medicinal importance and applications in the incised valleys of the Karakorum and Himalayan mountain ranges. Secondly, different ethnic groups in the upper (i.e., Hunza, Astore, Gilgit and Baltistan) and lower parts (Abbottabad, Haripur, Murree, Mansera, etc.) of north Pakistan possess different ethnoecological and traditional knowledge, particularly about plant diversity.

### Wild food species

The reliance of indigenous peoples and local communities on plant resources account for up to 95 % of their survival requirements [53]. Among the potential uses of plants those related to medicine and food have central importance because they are essential to human survival. A remarkable diversity of using edible wild plant species exists among the inhabitants of Tormik valley concerning food selection. Twenty-three species including 8 species of edible wild fruits and 13 species of vegetables were reported to be used by the inhabitants of the area (Table 3). To our knowledge six species of vegetables, including *Cerastium fontanum* Baumg.*, Cicer microphyllum* Royle.*, Onosma hispida, Pleurospermum candollei, Scorzonera hondae* Kitam. and *Silene vulgaris* (Moench) Garcke. and two species of edible wild fruits, i.e. *Chenopodium foliosum* and *Cousinia thomsonii* C. B. Clarke. have never been reported before from the study area and its surroundings. However, the rest of the botanical taxa were similar to those reported previously [13, 54]. Inhabitants of the valley try to derive benefit from the available species either for a change of taste or to fulfill their nutritional requirements unintentionally, as they do not have nutritionists nor do they care about it. This is also rationalized by [55], but the long-term utilization of wild plants is threatened due to development of edible foods markets.

### Cultural aspects of botanical taxa

The results presented in Table 4 demonstrate that 34 plant species have various indigenous uses among local peoples according to their cultural requirements (Fig. 2). Inhabitants of Tormik Valley use 11 plant species as fodder and forage. Grass species such as *Bromus pectinatus* Thunb. and *Poa pratensis* L. are cut and fed to livestock in fresh condition or stored for the winter season when vegetation totally vanishes in the valley. *Ribes alpestre* wall. exDecne.*, Rosa* spp. *Spiraea canescens* D.Don and *Tamaricaria elegans* (Royle) Qaiser & Ali are used in making huts and fencing. Hut construction is a common tradition in mountainous regions which are principally used in summer to rear livestock. The twigs, branches and stems of these species are used to build huts. Moreover, fences are built around home gardens, vegetable patches and other crop fields to protect them from herbivorous animals. *Betula utilis, Juniperus* spp. *Lonicera heterophylla* Decne. and *Tamaricaria elegans* are among the species used as domestic fuel for cooking purposes. Locals keep goats and sheep as an essential part of their life. The wool and hair (of sheep and goats) are woven to create different clothes, local blankets and carpets. To this end, people use *Betula utilis, Juglans regia* L.*, Lonicera heterophylla* L. and *Spiraea canescens* to make the tools required for weaving. The flowers of *Aster himalaicus* C.B. Clarke.*, Betula utilis, Hylotelephium ewarsii* (Ledeb.) H.Ohba.*, Papaver nudicaule* L. and *Trifolium repens* L. are kept in homes and held in the hand for fragrance. Flowers of affection are mostly given by shepherds/pastoralist to their loved ones and villagers with ice when there is no ice in the main valley.

**Table 4:** Food and other non-medicinal plant uses recorded in Tormik Valley, Karakorum

Plant species/family/voucher number	Local name	Cultural uses													No. of uses
		V	F	FF	HF	DW	WW	FA	B	AT	T	PS	BR	ER	
*Artemisia brevifolia* Wall. Asteraceae QAU127152	Bustae	-	-	-	-	+	-	-	-	-	-	-	-	-	1
*Aster himalaicus* C.B. Clarke Asteraceae QAU127157	Ghzima	-	-	+	-	-	-	+	-	-	-	-	-	-	2
*Astragalus frigidus* A.Gray Fabaceae QAU127249	Shashal	-	-	+	-	-	-	-	-	-	-	-	-	-	1
*Astragalus psilocentros* Fisch. Fabaceae QAU127251	BiowaCharchu	-	-	-	-	-	-	-	-	-	-	-	+	-	1
*Berberis pseudumbellata* R. Parker Berberidaceae QAU127186	Skiorbu	-	-	-	-	-	-	-	-	-	+	-	-	-	1
*Betula utilis* D.Don. Betulaceae QAU127187	Staqpa	-	-	-	-	+	+	+		+	+	+	+	-	7
*Bromus pectinatus* Thunb. Poaceae QAU127133	Troyuk	-	-	+	-	-	-	-	-	-	-	-	-	-	1
*Cerastium fontanum* Baumg. Caryophyllaceae QAU27207	Bloghar	+	-	+	-	-	-	-	-	-	-	-	-	-	2
*Chenopodium album* L. Amaranthaceae QAU127212	Snio	+	-	+	-	-	-	-	-	-	-	-	-	-	2
*Chenopodium foliosum* Asch. Amaranthaceae QAU127215	SpangOsae	-	+	-	-	-	-	-	-	-	-	-	-	-	1
*Cicer microphyllum* Royle. Fabaceae QAU127253	Stranjung Stwa	+	-	-	-	-	-	-	-	-	-	-	-	-	1
*Codonopsis clematidea* C.B. Clark Campanulaceae QAU127203	Loo sunma	-	-	+	-	-	-	-	-	-	-	-	-	-	1
*Colutea paulsenii* Freyn*.* Fabaceae QAU127257	Rbana	-	-	-	-	-	-	-	-	-	+	-	-	-	1
*Convolvulus arvensis* L. Convolvulaceae QAU127220	Thringthring-mo	+	-	-	-	-	-	-	-	-	-	-	-	-	1
*Cousinia thomsonii* C. B. Clarke. Asteraceae QAU127162	Charchu	-	+	-	-	-	-	-	-	-	-	-	-	-	1
*Datura fastuosa* L. Solanaceae QAU127296	Datura	-	-	-	-	-	-	-	-	-	-	-	-	+	1
*Fragaria nubicola* Lindl. ex Lacaita Rosaceae QAU127282	KarochaeMarochae	-	+	-	-	-	-	-	-	-	-	-	-	-	1
*Geranium pratense* L. Geraniaceae QAU127234	PorStwa	-	-	+	-	-	-	-	-	-	-	-	-	-	1
*Hippophae rhamnoides* ssp. *turkestanica* L. Eleagnaceae QUA127225	Karsoq	-	+	-	+	-	-	-	-	-	-	-	-	-	2
*Hylotelephium ewarsii* (Ledeb.) H.Ohba Crassulaceae QAU127221	Gongchu	-	-	-	-	-	-	+	-	-	-	-	-	-	1
*Juglans regia* L. Juglandaceae QAU127311	Starga	-	-	-	-	-	+	-	-	-	-	-	-	-	1
*Juniperus communis* L. Cupressaceae QAU127122	Oshuk	-	-	-	-	+	-	-	-	-	-	-	-	-	1
*Juniperus excelsa* M. Bieb. Cupressaceae QAU127223	Shukpa	-	-	-	-	+	-	-	-	-	-	-	-	+	2
*Lonicera heterophylla* Decne. Caprifoliaceae QAU127205	Said	-	-	-	-	+	+	-	-	+	-	-	-	-	3
*Lotus corniculatus* L. Fabaceae QAU127254	Spangol	-	-	+	-	-	-	-	-	-	-	-	-	-	1
*Mentha royleana* Wall. Lamiaceae QAU127239	Foling	+	-	-	-	-	-	-	-	-	-	-	-	-	1
*Onosma hispida* Wall. & G. Don. Boraginaceae QAU127193	Kangmar	+	-	-	-	-	-	-	-	-	-	-	-	-	1
*Papaver nudicaule* L. Papaveraceae QAU127248	Nilo	-	-	-	-	-	-	+	-	-	-	-	-	-	1
*Pimpinella diversifolia* DC. Apiaceae QAU127140	Kohniod	+	-	-	-	-	-	-	+	-	-	-	-	-	2
*Pleurospermum candollei* (DC.) Benth. ex C. B. Clark. Apiaceae QAU127141	Braq Shandun	+	-	-	-	-	-	-	-	-	-	-	-	-	1
*Poa pratensis* L. Poaceae QAU127137	Rastwa	-	-	+	-	-	-	-	-	-	-	-	-	-	1
*Potentilla salesoviana* Steph. Rosaceae QAU127284	SniarmaStwa	-	-	-	-	-	-	-	-	-	-	-	+	-	1
*Prunus armeniaca* L. Rosaceae QAU127312	Chuli	-	-	-	-	-	-	-	-	-	-	+	-	-	1
*Rhododendron hypenanthum* Balf.f. Ericaceae QAU127226	Sursur	-	-	-	-	-	-	-	+	-	-	-	-	-	1
*Ribes alpestre* wall. ex Decne. Grossulariaceae QAU127236	Skioruru	-	+	-	+	-	-	-	-	-	-	-	-	-	2
*Rosa brunonii* Lindl. Rosaceae QAU127286	SiaMarpho	-	+	-	+	-	-	-	+	-	-	-	-	-	3
*Rosa webbiana* Wall. Rosaceae QAU127287	SiaSarfo	-	+	-	+	-	-	-	-	-	-	-	-	-	2
*Rumax nepalensis* Spreng. Polygonaceae QAU127271	Rashona	+	-	-	+	-	-	-	-	-	-	-	-	-	2
*Scorzonera hondae* Kitam. Asteraceae QAU127177	SkiniSmaghra	+	-	-	-	-	-	-	-	-	-	-	-	-	1
*Silene vulgaris* (Moench) Garcke. Caryophylaceae QAU127209	Bostwa	+	-	-	-	-	-	-	-	-	-	-	-	-	1
*Solanum nigrum* L. Solanaceae QAU127298	Drumba shokhlo	-	+	-	-	-	-	-	-	-	-	-	-	-	1
*Spiraea canescens* D.Don Rosaceae QAU127289	Skhsi	-	-	-	+	-	+	-	-	+	+	+	-	+	6
*Tamaricaria elegans* (Royle) Qaiser & Ali Tamaricaceae QAU127305	Ongbu	-	-	-	+	+	-	-	-	-	-	-	-	-	2
*Taraxacum officinale* F.H.Wigg Asteraceae QAU127181	Khosmas	+	-	-	-	-	-	-	-	-	-	-	-	-	1
*Trifolium pratense* L. Fabaceae QAU127259	Ol	-	-	+	-	-	-	-	-	-	-	-	-	-	1
*Trifolium repens* L. Fabaceae QAU127258	Hltabuksuk	-	-	+	-	-	-	+	-	-	-	-	-	-	2
*Urtica dioica* L. Urticaceae QAU127308	Khaeshing	+	-	-	-	-	-	-	-	-	-	-	-	-	1

*V* wild edible vegetable, *F* wild edible fruit, *FF* fodder and forage, *HF* hut and fencing, *DW* domestic wood, *WW* wool weaving, *FA* fragrance and affection, *B* beverages, *AT* agricultural tools, *T* thatching, *PS* polo stick, *BR* bio-propellant, *ER* evil repellant

The leaves, stems and roots of *Pimpinella diversifolia, Rhododendron hypenanthum* Balf.f. and *Rosa brunonii* Lindl. are used in making beverages like tea and coffee. *Betula utilis, Lonicera heterophylla, Spiraea canescens* are used to make domestic and agricultural tools like ploughs and field planning tools, as well as the handles of shovels, spades, axes, etc. The small twigs and branches of *Berberis pseudumbellata* R.Parker*, Betula utilis, Colutea paulsenii* Freyn*.* and *Spiraea canescens* are used to thatch different baskets and grass carriers (locally called *Chorong*). Baskets of different sizes and shapes are mostly used to collect fruits from trees to protect them from squeezing/pressing and for transportation. Three plant species are used for making polo (a common game in the region) sticks which are used as well as sold to the people of surrounding areas. The branches of *Potentilla salesoviana* Steph are stuck in the holes of house walls to deter or prevent the entrance of rats, snakes, insects, centipedes, millipedes and other arthropods. The perception of locals about ailments of unknown origin involves the presence of evil, and *Juniperus excelsa* is used as an evil repellant. Our findings of the cultural uses of plants have few comparisons in adjacent regions; nevertheless these findings can be compared with a few other studies from the Himalayas [12, 21, 43].

### Highly utilized species

Though the numbers of uses mentioned are fairly low, but they are sufficient to indicate that human beings try to fulfill their daily life necessities from easily available and abundant species. This is supported by the different uses of the valley natives, who try to utilize resources from forest dominant plant species. For example *Betula utilis* shows seven different ways of utilization. This subalpine tree, is thus under severe anthropogenic pressure [56, 57]. Among other species *Berberis psuedumbellaeta, Juniperus excelsa, Pimpinella diversifolia* and *Spiraea canescens* also have four uses. These species are also described as exploited in other areas of the Himalayas in Pakistan [58, 59]. *Hippophe rhamnoides, Juniperus communis, Rosa brunonii* Lindl and *Rosa webbiana* Wall were used in three different ways.

The growth habit and body form of plant species are of great interest to people when exploring different utilization techniques. The results showed that species with a robust body form, i.e. shrubs and trees, are under more human pressure than herbaceous soft bodied species. As *Trifolium repens, Solanum nigrum, Rumex nepalensis* Spreng.*, Ribes alpestre, Pleurospermum candollei, Onosma hispida, Mentha royleana* Wall.*, Lonicera heterophylla, Cousinia thomsonii, Convolvulus arvensis, Cicer microphyllum, Chenopodium album, Cerastium fontanum, Astragalus frigidus* A.Gray and *Urtica dioica* L. have just two uses each and another 40 species only a single use.

### Traditional medicine: a hope for mountain dwellers

Plants have been vital sources of curative traditional medicine preparations for human beings since ancient times [4, 60]. According to the World Health organization (WHO), Traditional medicine is any “health practice, approaches, knowledge and beliefs incorporating plant, animal and mineral based medicines, spiritual therapies, manual techniques and exercises applied to treat, diagnose and prevent illnesses” [61]. It is an undeniable fact that forest inhabitants have an intimate relationship with the indigenous flora and maintain immense knowledge on the uses of various forest products over centuries [45, 61] and struggle to meet their life necessities from them. For the people of remote areas herbal remedies are easily obtainable and effective drugs for treating their health issues. Therefore, they intentionally, as well as unintentionally, transfer their invaluable indigenous knowledge from one generation to the next orally without any written text.

### Implications for public health and environmental policies

From the results provided in Table 3 it is clear that stomach related health problems (ulcers, constipation, GIT infections, jaundice), and skin diseases (dermatitis) are the most prevalent health problems in the area. Stomach disorders are likely due to malnutrition and unhygienic food utilization. Skin problems can be attributed to the high altitude of the study area, where radiation from the sun tends to be more intense and potentially mutagenic. People traditionally treat such diseases with food-medicines, which in many cases are quite effective. Hence, the present findings provide very important insights for public-health officials, to formulate health policies taking into account the common health issues and Traditional Medicine (TM) practiced by the local people as part of their primary healthcare.

## Conclusion

The present study revealed that the valleys in the Karakorum Mountains in Northern Pakistan support a notable Traditional Knowledge on the local plants. Wild food plants have represented the milestone of the traditional food systems and could still represent a pillar of the local food sovereignty, while medicinal plants play a vital role, which need to be reconsidered and carefully re-evaluated by ethnopharmacologists and public health actors. The collected data may be also of interest to initiatives aimed at fostering sustainable rural development in an area that faces serious economic problems, widespread illiteracy, and isolation. The findings of this paper advocate the need for comprehensive trans-disciplinary researches aimed to ensure the dynamic conservation of invaluable local knowledge systems, as well as plant diversity in Pakistani mountain regions.
